# Effects of enriched seafood sticks (heat-inactivated *B. animalis* subsp. *lactis* CECT 8145, inulin, omega-3) on cardiometabolic risk factors and gut microbiota in abdominally obese subjects: randomized controlled trial

**DOI:** 10.1007/s00394-022-02904-0

**Published:** 2022-05-28

**Authors:** Judit Companys, Lorena Calderón-Pérez, Laura Pla-Pagà, Elisabet Llauradó, Berner Andrée Sandoval-Ramirez, Maria José Gosalbes, Ainara Arregui, Maddi Barandiaran, Antoni Caimari, Josep Maria del Bas, Lluís Arola, Rosa M. Valls, Rosa Solà, Anna Pedret

**Affiliations:** 1 Eurecat, Centre Tecnològic de Catalunya, Unitat de Nutrició i Salut, Avinguda Universitat, 1, 43204 Reus, Spain; 2 Functional Nutrition, Oxidation, and Cardiovascular Diseases Group (NFOC-Salut), Facultat de Medicina i Ciències de la Salut, Universitat Rovira i Virgili, C/Sant Llorenç 21, 43201 Reus, Spain; 3 Fundación de la Investigación Sanitaria y Biomédica, Valencia, Spain; 4 CIBERESP, Madrid, Spain; 5 Angulas Aguinaga Research Center, Laskibar bailara, 5, 20271 Irura, Gipuzkoa Spain; 6 Centre Tecnològic de Catalunya, Biotechnology Area, Reus, Spain; 7 Nutrigenomics Research Group, Faculty of Chemistry, Universitat Rovira i Virgili, Tarragona, Spain; 8 Hospital Universitari Sant Joan de Reus, Reus, Spain

**Keywords:** Cardiometabolic disease, Gut microbiota, Postbiotics, Inulin, Omega 3, Type 2 diabetes management

## Abstract

**Purpose:**

To assess the effects of enriched seafood sticks with postbiotic and bioactive compounds on CMD risk factors and the gut microbiota in abdominally obese individuals.

**Methods:**

Randomized, double-blind, parallel, placebo-controlled trial with abdominally obese individuals. Participants (*n* = 120) consumed 50 g/day of enriched seafood sticks containing SIAP: (10^10^ colony forming units (CFUs) of heat-inactivated *B. animalis* subsp. *lactis* CECT8145, 370 mg/day omega 3 and 1.7 g/day inulin), or 50 g/day of placebo seafood sticks for 12 weeks. At 12 weeks, an acute single-dose study of 4 h was performed.

**Results:**

Sustained SIAP2 consumption significantly decreased the insulin by − 5.25 mg/dL and HOMA-IR (homeostatic Model Assessment of Insulin Resistance) by − 1.33. In women, SIAP2 consumption significantly decreased the pulse pressure (PP) by − 4.69 mmHg. Gut microbiota analysis showed a negative association between glycemic parameter reduction and *Alistipes finegoldii* and Ruminococcaceae, and between PP reduction and *Prevotella 9-*ASV0283 and Christensenellaceae. In the acute single dose-study 4-h, SIAP2 consumption produced a lower increase in the postprandial circulating triglyceride levels [23.9 (7.03) mg/dL (mean [standard error])] than the observed with placebo [49.0 (9.52)] mg/dL.

**Conclusion:**

In abdominally obese individuals, enriched seafood sticks induce a potential protection against type 2 diabetes development by the reduction in the insulin and HOMA-IR; and in cardiovascular disease, in women, by the PP reduction. These effects are accompanied by partial changes in the gut microbiota composition. The enriched seafood sticks reduce the atherogenic triglyceride postprandial concentrations. Our results support the use of enriched seafood sticks as a complementary strategy in the management of CMD risk factors.

**Registration number of Clinical Trial:**

(www.ClinicalTrials.gov): NCT03630588 (August 15, 2018).

**Supplementary Information:**

The online version contains supplementary material available at 10.1007/s00394-022-02904-0.

## Introduction

Cardiovascular diseases (CVDs) are the most common cause of death globally [1]. CVDs are included in denominated cardiometabolic diseases (CMDs), along with diabetes mellitus and chronic renal failure, which together substantially contribute to morbidity and mortality on a global scale [2]. One of the major CMD risk factors to consider is abdominal obesity, defined by the World Health Organization (WHO) Expert Consultation on Obesity, which stated that a waist circumference (WC) of at least 102 cm in men and 88 cm in women is associated with a substantially increased risk of CMD [3] and exhibits a strong positive association with prediabetes [4, 5], and elevated serum lipids, including serum triglyceride (TG) concentration and high blood pressure (BP) in adults [6].

Probiotics, defined as live microorganisms that confer a health benefit to the host when administered in adequate amounts [7], have shown beneficial effects against CMDs. In this sense capsule/powder probiotic supplementation could reduce anthropometric parameters, and contribute to type 2 diabetes (T2D) management [8]. In particular, the effects of the probiotic *Bifidobacterium animalis* subsp. lactis CECT 8145, also named BPL1 in its live form, has already been evaluated in randomized controlled trials (RCTs), which have shown a reduction in biomarkers of anthropometric adiposity, including the visceral fat area in subjects with abdominal obesity. Moreover, a preclinical study also showed improvements in the lipid profile and insulin sensitivity in cafeteria-fed obese rats [9]. In contrast, postbiotics, defined as non-living microorganisms with probiotic effects [10], have also shown beneficial effects on CMDs. In this sense, the heat-treated BPL1™ (BPL1™ HT) has similarly demonstrated reductions in biomarkers of anthropometric adiposity, particularly the visceral fat area in subjects with abdominal obesity, but this form yields better results than those obtained with the live/activated form [11–13]. Probiotics and postbiotics are often administered with other bioactive compounds to achieve better effects on different diseases based on the synergy between the compounds. As an example, the combination of a probiotic (*Lactobacillus sporogenes*) with inulin exerts beneficial effects on the TG and high-density lipoprotein (HDL) cholesterol levels in humans [14]. Omega-3 fatty acids are another bioactive compound that has been studied in combination with probiotics and postbiotics, and the combination of the probiotic VSL#3 with omega-3 reduces the total cholesterol and TG levels and increases HDL cholesterol levels [15] in overweight adult subjects. Additionally, Omega-3, eicosapentaenoic acid (EPA) and docosahexaenoic acid (DHA), have several accepted health claims involving benefits on cardiovascular risk factors (TG and BP levels) [16] in humans. However, in postprandial state, the reduction of the atherogenic TGs increase observed by omega-3 and/or postbiotic consumption is a new challenge [17].

Among the different dietary strategies for combating CMDs, the dietary matrices that incorporate probiotics, postbiotics or other bioactive compounds must be considered, whereas high-quality RCTs comparing the effectiveness of adding probiotics to different food matrices remain scarce. In this context, the most studied food matrix for incorporating probiotics or postbiotics is dairy products (milk and yogurt) [18], and other food matrices have scarcely been exploited by the food industry, such as fish, which naturally contain the bioactive compounds EPA and DHA.

Importantly, the gut microbiota has been identified as the principal mechanism of action through which probiotics exert benefecial effects on CMDs [19]. Probiotics and postbiotics could modulate the abundance of different bacterial species in the gut microbiota that could have an impact on health, for example, by reducing the Firmicutes/Bacteroidetes ratio [20], whereas postbiotic impact on CMDs are still unknown.

Based on all the above mentioned factors, the present study aimed to assess the sustained and acute effects of white fish-based seafood sticks enriched with heat-treated *Bifidobacterium animalis* subsp. *lactis* BPL1™ as a postbiotic and inulin, with EPA and DHA as bioactive compounds, denominated SIAP2, on CMD risk factors by modifying gut microbiota in subjects with abdominal obesity.

## Materials and methods

### Study population

Subjects were recruited between August 2018 and December 2018 from general databases of participants from previous studies and via media and social networks. Eurecat, Centre Tecnològic de Catalunya, Unitat de Nutrició i Salut has its own profile and experience in the recruitment of participants for clinical trials. The subjects’ inclusion criteria were as follows: (a) be aged > 18 years; (b) the male and the female subjects had a WC ≥ 102 cm and ≥ 88 cm, respectively, in agreement with the European guidelines [21]; (c) all the patients signed the informed consent form. Exclusion criteria were as follows: (a) having any condition incompatible with magnetic resonance imaging (MRI) tests, such as metallic implants sensitive to magnetic fields, pacemakers or suffering from claustrophobia; (b) glucose > 126 mg/dL; (c) body mass index (BMI) ≥ 40 kg/m^2^; (d) WC > 150 cm; (e) dyslipidemia (low-density lipoprotein [LDL] cholesterol ≥ 189 mg/dL and/or TG ≥ 350 mg/dL); (f) use of medications, antioxidants, vitamin supplements or phytotherapeutic products that can interfere with the study intervention; (g) chronic alcoholism; (h) chronic gastrointestinal disorders; (i) intolerances and/or food allergies related to the study product; (j) anemia (hemoglobin ≤ 13 mg/dL in men and ≤ 12 mg/dL in women); (k) chronic disease with clinic manifestation; (l) pregnant or intending to become pregnant or breastfeeding period; (m) following a hypocaloric diet and/or undergoing pharmacological treatment for weight loss; (n) eating behavior disorders; (o) current or past participation in a clinical trial within the last 3 months.

Adverse effects were registered at each visit according to the Medical Dictionary for Regulatory Activities (MedDRA dictionary, MedDRA version 19.0, English, March 2016). Furthermore, the subjects were asked about their symptoms or any discomfort at each visit. All the subjects signed an informed consent form prior to participation in the study. The protocol was approved by the Clinical Research Ethical Committee of the HUSJ, Reus, Catalonia, Spain (Ref. CEIm: 086/2017).

The protocol and trial were conducted in accordance with the Helsinki Declaration and Good Clinical Practice Guidelines of the International Conference of Harmonization (ICH GCP) and reported CONSORT criteria. The trial was registered at ClinicalTrials.gov with the identifier number: NCT03630588.

### Intervention product

The study products were seafood sticks based on two fish species, Alaska pollock and Pacific hake, and were manufactured by Angulas Aguinaga (Spain). As described in Table 1, the intervention products were as follows: (1) SIAP2, 50 g/day conventional seafood sticks + heat-treated *Bifidobacterium animalis* subsp. *lactis* CECT 8145 (10^10^ CFU/100 g of seafood sticks) + 370 mg/day EPA and DHA + 1.7 g/day inulin; and (2) placebo, 50 g/day conventional seafood sticks. The isolation and growth of *Bifidobacterium animalis* subsp. *lactis* CECT 8145 has been described previously [9].

**Table 1:** Nutritional composition of seafood sticks enriched with postbiotic and bioactive compounds (SIAP2) and conventional seafood sticks (Placebo)

Component	SIAP2 dose day (50 g)	Placebo dose day (50 g)
Energy (kcal)	47.50	45.00
Total fat (g)	1.80	1.65
Saturated fat (g)	0.30	1.00
Monounsaturated fat (g)	0.60	0.50
Polyunsaturated fat (g)	0.90	0.10
Omega 3 (EPA + DHA) (mg)	370.00	N.A
Total carbohydrates (g)	3.60	3.85
Sugars (g)	1.30	1.45
Dietary fibre (inulin) (g)	1.70	N.A
Proteins (g)	4.00	4.00
Salt (g)	0.90	0.90

*EPA*, eicosapentaenoic acid; *DHA*, docosahexaenoic acid; *N.A.*, non available

Both products were administered in identical seafood sticks and were similar in appearance and smell and only differentiated by a code (111 or 222) assigned by an independent researcher who was not related to the study to guarantee blinding.

### Study design

A randomized, double-blind, parallel, placebo-controlled clinical trial including subjects with abdominal obesity was conducted for 12 weeks.

This study was conducted at Eurecat, Centre Tecnològic de Catalunya, Unitat de Nutrició i Salut, in Reus, Catalonia, Spain. A total of 120 subjects were randomized into the following two groups: (1) placebo and (2) SIAP2 groups. Each day, each subject consumed three sticks (one sachet) corresponding to a total seafood stick weight of 50 g (SIAP2 or placebo). All subjects received their seafood sticks frozen in packs of 600 g (36 sticks packed in sachets of three bars), and the subjects picked up their seafood stick packages every 6 weeks. During this period, the subjects were asked to attend the following four visits: an initial screening visit (V0), an inclusion visit before starting the intervention (V1), a visit after 6 weeks of the intervention (V2) and, a visit on the last day of the intervention (after 12 weeks; V3). The subjects were asked to return all sachets of seafood sticks (empty or not empty) to the center to ensure registration of the daily amount of unconsumed product.

Moreover, an acute study was performed after 12 weeks of sustained consumption to evaluate wether sustained consumption of enriched seafood sticks can reduce the postprandial meal effects. This study was conducted at the final visit (V3) that lasted from 8:00 a.m. to 12:00 a.m. At baseline (0 h) and 2 and 4 h after consumption of a single dose (50 g) of seafood sticks (placebo or SIAP2) plus a high-fat meal, including 80 g of white bread, 30 g of hard cheese, 55 g of boiled egg, 30 g of refined olive oil and water ad libitum, blood samples were collected, and the BP was measured [22]. The design of the study is detailed in Supplementary Fig. S1.

The blood samples were stored at − 80 °C in the Biobanc of HUSJ’s central laboratory (biobanc.reus@iispv.cat) until use for batch analyses. Fecal samples were collected during the inclusion visit (V1) and at the final visit (V3).

### Randomization and blinding

The generated randomization sequence was prepared by an independent researcher who was unrelated to the study to guarantee blinding. A randomization sequence was created using the SAS 9.2 (Cary, NC, USA: SAS Institute Inc.) statistical software PROC PLAN based on a 1:1 ratio. The subjects were randomly allocated to one of two experimental groups, and the randomization list indicated the study product that would be consumed by each subject during the 12-week study period. An independent person who was unrelated to the study kept the blinding code. The randomization list remained hidden until the end of the experimental intervention and was revealed once the data registration and statistical analysis were completed. All investigators, staff, subjects and outcome assessors involved in the development of the trial remained blinded until the study and statistical analysis were completed.

The sample size was estimated assuming a type I error of 0.05 (two-sided) and at least 80% power for detecting a mean area of 5 cm^2^ in a between-group reduction of the visceral abdominal fat area (VFA), which is considered a major CMD risk factor, over 12 weeks, as an indicator of abdominal obesity [23]. Based on this estimation, 43 subjects were assigned to each group. The standard deviation was estimated to equal 13.02 cm^2^ (19.20). The standard deviation was estimated based on data from a previous clinical trial [24].

The data are expressed as the means ± standard deviations, averages (95% confidence intervals, CIs) or medians (25th–75th percentiles). The normality of each variable was determined by the Shapiro–Wilk test. An efficacy analysis was performed using the intention-to-treat (ITT) population. Intratreatment comparisons were made using a general linear model (GLM) with Bonferroni correction and the age, sex and body weight at the beginning of the study as covariables. Intertreatment comparisons were performed using the ANCOVA model with the age, sex and body weight at the beginning of the study as covariables. The level of significance was set to *p* < 0.05. The statistical analyses were performed using IBM SPSS version 22.

### Outcome measures

#### Primary outcome

The VFA, quantified by an MRI-based transverse body scan in one 5-cm axial slice over L5–S1 [25], was measured at V1 and V3. The MRI study was performed with a General Electric 3 Tesla HDXT MRI after 6 h of fasting.

#### Secondary outcomes

Anthropometric parameters were obtained at V0, V1, V2 and V3 while the subjects were wearing lightweight clothing and no shoes. Trained dietitians measured the body weight and body composition of the subjects using a body composition analyzer (Tanita SC 330-S; Tanita Corp., Barcelona, Spain) and the height of the subjects using a wall-mounted stadiometer (Tanita Leicester Portable; Tanita Corp., Barcelona, Spain). The WC was measured at the umbilicus using a 150-cm anthropometric steel measuring tape (McAuley PA, 2014). The WC (cm)/height (cm) and the conicity index (CI), defined as WC (m)/[0.109 X√ {body weight (kg)/height (m)}], were calculated.

The lipid profiles, such as total cholesterol, HDL cholesterol, LDL cholesterol, TGs, very-low density lipoprotein (VLDL), apolipoprotein A-1 (Apo A-1), apolipoprotein B-100 (Apo B-100), and non-esterified fatty acids (NEFAs), and glucose metabolism markers, such as insulin and glucose, were measured (at V1 and V3) in serum using standardized enzymatic automated methods with a Beckman Coulter–Synchron autoanalyzer (Beckman Coulter–Synchron, Galway, Ireland). The homeostatic model assessment index (HOMA-IR) was measured with the formula insulin x glucose/405.

LDL cholesterol was calculated with the Friedewald formula (Friedewald WT, 1972). In the acute study, the serum lipid profile and glucose metabolism markers were measured at baseline and 2 h and 4 h postprandial. The subcutaneous abdominal fat area was evaluated by an automatic MRI transverse body scan in one 5-cm axial slice over L5–S1 [25].

After the subjects rested for 2–5 min while seated, the systolic and diastolic BP were measured twice at a 1-min interval using an automatic sphygmomanometer (OMRON HEM-907; Peroxfarma, Barcelona, Spain) by a physician. The mean values were employed for the statistical analyses. These parameters were measured at each visit in the sustained study (V0, V1, V2 and V3) and at baseline and 2 h and 4 h postprandial in the acute study. The pulse pressure (PP), which represents the force that the heart generates each time it contracts, was determined by the difference between the systolic and diastolic BP readings [26]. This parameter was measured at each visit in the sustained study (V0, V1, V2 and V3) and at baseline and 2 h and 4 h postprandial in the acute study.

### Dietary and physical activity assessment

Throughout the study period, the subjects were encouraged to maintain their lifestyle, such as physical activity, and follow the dietary recommendations for cardiovascular health provided according to guidelines of the 2013 Adult Treatment Panel (ATP III) [27]. Moreover, the subjects were asked to avoid the consumption of probiotics and any antibiotic treatment.

For evaluation of the subjects’ diet, all the subjects were asked to complete a 3-day dietary record (two weekdays and one weekend day) at the baseline visit, dietitians checked the dietary record with the subjects, and in the case of missing product quantities, the dieticians used a portion book to complete the record. Another 3-day dietary record was completed at the final visit to assess the dietary macro- and micronutrient intake. The mean daily intake of energy and nutrients was calculated with Spanish Food Composition Tables [28] using computerized software (PCN Pro 1.0).

At baseline and after 6 and 12 weeks of intervention, the subjects underwent a physical examination by a general practitioner and completed a Physical Activity Questionnaire (Class AF) [29].

### Fecal microbiota DNA extraction and sequencing

On visits V1 and V3, all subjects were asked to collect a fecal sample, in a sterile plastic container with 10 mL of RNAlater^®^ storage solution (Sigma-Aldrich Quimica SL; Madrid, Spain) no later than 72 h before the visit, using a stool collection device (Protocult™ ABC, Minnesota, EEUU). Immediately after delivery, stools were stored at − 80 °C until DNA extraction.

Fecal samples were diluted with Phosphate-Buffered Saline (PBS) solution (dilution 1:2). Subsequently, fecal samples were centrifuged at 200 rpm at 4 °C for 5 min to remove debris. A robotic workstation (The MagNA Pure LC Instrument, Roche) extracted total DNA from pelleted bacterial cells using the MagNA Pure LC DNA isolation kit III (Bacteria, Fungi) (Roche) according to the manufacturer’s instructions. The region V3–V4 of the 16S rRNA gene was amplified by PCR and used to amplicon library construction following Illumina instructions. Sequencing was performed with the Kit V3 (2 × 300 cycles) in MiSeq platform (Illumina, Eindhoven, Netherlands) in the Centre for Public Health Research (FISABIO-Salud Pública, Valencia, Spain). All the sequences have been deposited in the EBI database under the number PRJEB36385.

### Microbiota sequence analysis

We applied Prinseq (v0.20.4) [30] for trimming the ends of each read with bases with quality lower than 30 and discarding reads shorter than 100 bases. The following steps were performed with R (v3.6.0) [31] by means of the corresponding functions of the DADA2 library (v1.8.0) [32]. Dereplication was carried out to combine all identical reads into unique sequences, with abundance equal to the number of reads combined, and error estimation was considered; after these steps, amplicon sequence variants (ASVs) were inferred. ASVs which map against the human genome were removed to discard possible contaminations. ASVs were taxonomically classified using Silva database (version 123) [33]. This classification can reach a resolution of genus level, but exact matching (100% identity) is applied to assign a unique species to each ASV sequence. Additionally, ASVs with an assigned genus but without exact matching at the species level were mapped against the same reference database with a minimum of 97% of identity.

Alpha diversity parameters, Shannon diversity index and Chao1 richness estimator, were calculated using Vegan library in R package. The compositional differences between communities (beta-diversity) were assessed based on Bray–Curtis dissimilarity index in Principal Coordinates Analysis (PCoA). To determine the contribution of an environmental factor to the variability of the microbiota composition between groups, we performed ADONIS test using R package.

To detect ASVs as biomarkers, a linear discriminant analysis (LDA) with linear discriminant effect size (LEfSe) algorithm was performed. α-Value < 0.05 was fixed and the threshold used to consider a discriminative feature for the logarithmic LDA score was set at > 2.0 or > 2.5.

The associations between dietary nutrients or clinical variables and microbiota composition, at ASV level, was evaluated using sparse partial least square (sPLS), a multivariant method implemented in ‘mixOmics’ R package [34]. The inner product of the coordinates of each variables is an approximation of their association score. This threshold was seated at 0.5 to represent the relationships in the networks.

## Results

**Fig. 1:**
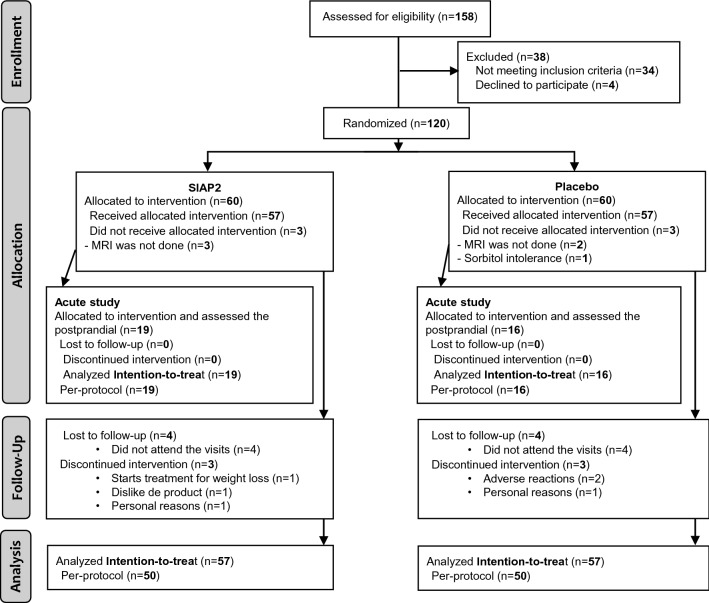
Flowchart of the participant study

Of 158 subjects who were assessed for eligibility between August 2018 and December 2018, 38 did not meet the inclusion criteria and were excluded. The remaining 120 subjects were randomly allocated to the SIAP2 group (*n* = 60) or placebo group (*n* = 60). Of all the subjects, 57 in each group were administered their allocated intervention. For the acute study at the final visit (V3), 19 subjects were enrolled in the SIAP2 group, and 16 subjects were included in the placebo group. The subjects who were screened, enrolled, and lost to follow-up during treatment and analysis are shown in Fig. 1. No differences in baseline characteristics and in dietary intake were observed among intervention groups (Supplementary Item 1, Supplementary Item 2).

### Variable outcomes

#### Anthropometric measures

The VFA and subcutaneous abdominal fat area changes stratified by gender are shown in Table 2. After 12 weeks of intervention, no intra- or intertreatment differences in either the visceral, subcutaneous or in the ratio visceral/subcutaneous fat area were observed in the analysis of all the samples or in the gender-based subgroup analyses (*p* > 0.05).

**Table 2:** Changes in visceral and subcutaneous fat areas by gender in abdominally obese subjects after supplementation with placebo or SIAP2 at 12 weeks of intervention

	SIAP2 (*n* = 57)	Placebo (*n* = 57)	SIAP2 vs. Placebo		Male				Female			
					SIAP2	Placebo	SIAP2 vs. Placebo		SIAP2	Placebo	SIAP2 vs. Placebo	
	MD (95% CI)	MD (95% CI)	MD	*p* value	MD (95% CI)	MD (95% CI)	MD	*p* value	MD (95% CI)	MD (95% CI)	MD	*p* value
*Visceral fat area (cm*^*2*^*)*												
Δ Week 12-Baseline	− 0.44 (− 6.50; 5.62)	− 2.76 (− 8.87; 3.35)	2.32	0.594	− 3.27 (− 12.3; 5.76)	− 5.88 (− 14.9; 3.14)	2.61	0.685	3.43 (− 4.09; 10.9)	2.41 (− 5.28; 10.1)	1.01	0.851
*Subcutaneous fat area (cm*^*2*^*)*												
Δ Week 12-Baseline	− 3.98 (− 11.3; 3.40)	− 0.34 (− 0.74; 6.74)	− 3.63	0.485	− 8.37 (− 18.7; 1.92)	− 1.01 (− 10.3; 8.31)	− 7.35	0.295	2.37 (− 8.51; 13.2)	0.69 (− 10.8; 12.2)	1.68	0.832
*VAT/SAT ratio*												
Δ Week 12-Baseline	0.011 (− 0.05; 0.03)	− 0.008 (− 0.04; 0.03)	− 0.003	0.916	− 0.020 (− 0.08; 0.04)	− 0.015 (− 0.07; 0.04)	− 0.005	0.897	0.001 (− 0.03; 0.03)	0.004 (− 0.03; 0.03)	− 0.003	0.899

Data expressed as mean (95% confidence interval, CI)

*VAT*, visceral adipose tissue; *SAT*, subcutaneous adipose tissue; *MD*, mean difference

ANCOVA Model adjusted by sex, age, basal values, and physical activity at the beginning of the study. *p < 0.05

The changes in body weight, BMI, WC, WC/height ratio and Conicity index at weeks 6 and 12, and stratified by gender are shown in Supplementary item 3. After 6 weeks of intervention, the body weight was reduced in the SIAP2 group, and this decrease was significantly different from the changes observed in the placebo group (*p* = 0.030). Moreover, a decrease in the WC and in the WC/height ratio was observed after 6 weeks of intervention (*p* < 0.05) in both groups, and no differences were found between the groups.

The gender-based analysis of the data revealed that the decrease in the WC remained significant in the placebo group (*p* < 0.05) and that the decrease in the WC/height ratio remained significant after both treatments (*p* < 0.05). In addition, a decrease in the Conicity index was observed after the placebo treatment in men (*p* < 0.05), and no intertreatment differences were observed.

After 12 weeks, no intra- or intertreatment differences in the anthropometric or adiposity variables evaluated were observed in the analysis of all the samples or in the gender-based subgroup analyses.

#### Blood pressure and pulse pressure

The changes in systolic and diastolic BP and PP measures at weeks 6 and 12 are detailed in Table 3. No intra- or intertreatment differences in vascular parameters were observed after either 6 weeks or 12 weeks of intervention. The gender-based analysis of the data revealed decreases in systolic BP and PP in women after 6 weeks of SIAP2 treatment. These decreases were significant compared with the changes observed after placebo treatment (*p* < 0.05). In women, the decrease in PP of − 4.69 mmHg found in the SIAP2 group after 12 weeks of intervention remained significantly different from that found in the placebo group (*p* = 0.046). In the acute study, both groups had reduced systolic BP at 2 h, but the effect reached significance only in the placebo group, and diastolic BP was significantly reduced in both groups (*p* < 0.05); in addition, no changes in PP in either group and no differences between the treatments were observed, as described in Table 4.

**Table 3:** Changes in blood pressure parameters from baseline in abdominally obese subjects after supplementation with placebo or SIAP2 for 6 and 12 weeks

	SIAP2 (*n* = 57)	Placebo (*n* = 57)	SIAP2 vs. Placebo		Male				Female			
					SIAP2	Placebo	SIAP2 vs. Placebo		SIAP2	Placebo	SIAP2 vs. Placebo	
	MD (95% CI)	MD (95% CI)	MD	*p* value	MD (95% CI)	MD (95% CI)	MD	*p* value	MD (95% CI)	MD (95% CI)	MD	*p* value
*SBP (mmHg)*												
Δ Week 6-Baseline	− 1.536 (− 4.17; 1.10)	− 0.268 (− 2.90; 2.36)	− 1.269	0.502	0.566 (− 2.78; 3.92)	− 0.477 (− 3.83; 2.87)	1.043	0.664	− 5.472 (− 9.38; − 1.56)*	0.745 (− 3.17; 4.65)	− 6.217	0.029*
Δ Week 12-Baseline	− 0.509 (− 3.24; 2.23)	− 1.679 (− 4.44; 1.08)	1.170	0.553	− 0.987 (− 4.36; 2.39)	− 2.072 (− 5.45; 1.31)	1.085	0.654	− 0.545 (− 5.06; 3.97)	− 0.293 (− 4.91; 4.32)	− 0.252	0.938
*DBP (mmHg)*												
Δ Week 6-Baseline	− 1.517 (− 3.41; 0.38)	− 1.144 (− 3.04; 0.75)	− 0.374	0.784	− 1.477 (− 3.55; 0.59)	− 1.978 (− 4.02; 0.06)	0.501	0.733	− 1.458 (− 5.00; 2.09	0.024 (− 3.60; 3.65)	− 1.482	0.561
Δ Week 12-Baseline	− 0.825 (− 2.61; 0.96)	− 1.568 (− 3.35; 0.21)	0.743	0.562	− 1.157 (− 3.28; 0.97)	− 1.348 (− 3.44; 0.75)	0.191	0.899	− 0.389 (− 3.61; 2.83)	− 1.866 (− 5.16; 1.43)	1.477	0.524
*PP (mmHg)*												
Δ Week 6-Baseline	− 0.308 (− 2.57; 1.95)	0.820 (− 1.42; 3.06)	− 1.127	0.485	1.330 (− 1.34; 4.00)	1.503 (− 1.12; 4.13)	− 0.174	0.927	− 4.091 (− 7.60; − 0.58)*	1.091 (− 2.42; 4.60)	− 5.183	0.042*
Δ Week 12-Baseline	− 0.582 (− 2.61; 2.09)	0.03 (− 2.37; 2.43)	− 0.973	0.562	− 0.354 (− 3.11; 2.40)	0.860 (− 3.57; 1.85)	0.596	0.796	− 1.921 (− 5.08; 1.23)	2.770 (− 0.53; 6.07)	− 4.690	0.046*

Data expressed as mean (95% Confidence Interval, CI).

*SBP*, systolic blood pressure; *DBP*, diastolic blood pressure; *PP*, pulse pressure

ANCOVA model adjusted by sex, age, basal values, and physical activity at the beginning of the study. *p < 0.05

**Table 4:** Acute changes in blood pressure, serum glucose, insulin and lipid profile after interventions

Variable	Baseline (0 h)	Change after intervention		
		2 h	4 h	P (trend)^a^
*Systolic blood pressure (mmHg)*				
Placebo (*n* = 16)	127 (4.44)	− 9.31 (3.07)*^†^	− 5.06 (3.56)	0.505
SIAP2 (*n* = 19)	128(2.52)	− 3.16 (3.09)	0.263 (2.43)	0.780
*Diastolic blood pressure (mmHg)*				
Placebo (*n* = 16)	79 (2.69)	− 7.81 (1.66)^‡,¥^	− 2.62 (1.74)	0.614
SIAP2 (*n* = 19)	81 (1.72)	− 4.42 (1.58)*^,¥^	− 0.158 (1.62)	0.397
*Pulse pressure (mmHg)*				
Placebo (*n* = 16)	48 (2.57)	− 1.50 (2.53)	− 2.44 (2.60)	0.714
SIAP2 (*n* = 19)	48 (1.96)	1.26 (2.27)	0.421 (1.62)	0.432
*Glucose (mg/dL)*				
Placebo (*n* = 16)	96 (2.43)	7.56 (3.76)	− 6.62 (2.51)	0.031^a^
SIAP2 (*n* = 19)	100 (2.04)	10.6 (3.79)^†,‡^	− 5.00 (2.01)	0.352^a^
*Insulin (mmol/L)*				
Placebo (*n* = 16)	11.7 (2.57)	21.9 (5.68)^†^	4.00 (1.92)	0.242^a^
SIAP2 (*n* = 19)	14.8 (2.50)	21.9 (4.93)^†^	4.89 (4.38)	*0.098*^a^
*Total cholesterol (mg/dL)*				
Placebo (*n* = 16)	206 (4.70)	− 4.50 (2.47)	− 2.69 (2.35)	0.609^b^
SIAP2 (*n* = 19)	210 (8.04)	− 8.47 (1.91)^†,‡^	− 2.32 (2.39)	0.214^b^
*HDL cholesterol (mg/dL)*				
Placebo (*n* = 16)	54.7 (2.45)	− 2.25 (0.66)*^,‡^	− 0.438 (0.60)	0.484^b^
SIAP2 (*n* = 19)	51.3 (2.49)	− 2.84 (0.51)^≠,⁋^	0.421 (0.72)	0.106^b^
*LDL cholesterol (mg/dL)*				
Placebo (*n* = 16)	126 (4.87)	− 10.0 (2.17)^†^	− 12.1(1.98)^†^	0.558^b^
SIAP2 (*n* = 19)	134 (6.32)	− 13.3 (1.62)^≠,‡^	− 7.63 (1.71)^†^	0.623^a^
*VLDL cholesterol (mg/dL)*				
Placebo (*n* = 16)	25.2 (3.48)	7.75 (1.58)^†^	9.87 (1.92)^†^	0.318^b^
SIAP2 (*n* = 19)	24.3 (2.44)	7.63 (1.27)^≠^	4.89 (1.41)^†^	0.391^b^
*Total cholesterol/HDL ratio*				
Placebo (*n* = 16)	3.98 (0.26)	0.066 (0.02)*^,‡^	− 0.019 (0.03)	0.662^b^
SIAP2 (*n* = 19)	4.28 (0.26)	0.044 (0.01)*^,‡^	− 0.104 (0.03)*	0.122^b^
*LDL/HDL ratio*				
Placebo (*n* = 16)	2.43 (0.17)	− 0.101 (0.02)^†,‡^	− 0.210 (0.03)^≠^	0.047^b^
SIAP2 (*n* = 19)	2.75 (0.20)	− 0.146 (0.03)^†^	− 0.193 (0.02)^≠^	0.014^b^
*Triglycerides (mg/dL)*				
Placebo (*n* = 16)	126 (17.3)	38.5 (8.06)^†^	49.0 (9.52)^†^	0.248^a^
SIAP2 (*n* = 19)	121 (12.2)	38.2 (6.31)^≠^	23.9 (7.03)*^, ¥^	0.472^b^
*Non-esterified fatty acids (mmol/L)*				
Placebo (*n* = 16)	0.50 (0.03)	− 0.153 (0.04)*^,‡^	− 0.066 (0.04)	0.023^a^
SIAP2 (*n* = 19)	0.46 (0.04)	0.001 (0.09)	0–013 (0.04)	0.864^b^
*Apo A-1 (mg/dL)*				
Placebo (*n* = 16)	153 (3.59)	− 3.25 (1.76)	− 2.81 (1.95)	0.354^a^
SIAP2 (*n* = 19)	145 (4.11)	− 3.89 (0.95)^†^	− 1.95 (1.61)	0.110^b^
*Apo B-100 (mg/dL)*				
Placebo (*n* = 16)	107 (4.56)	− 4.75 (1.33)*	− 4.31 (1.48)*	0.646^b^
SIAP2 (*n* = 19)	114 (6.82)	− 6.28 (1.01)^≠^	− 4.61 (1.26)^†^	0.201^b^
*Apo A-1/Apo B-100 ratio*				
Placebo (*n* = 16)	1.49 (0.09)	0.030 (0.01)^†^	0.033 (0.01)	0.630^a^
SIAP2 (*n* = 19)	1.33 (0.08)	0.042 (0.01)*	0.044 (0.01)^†^	0.703^b^

*Apo*, apolipoprotein; *HDL*, high density lipoproteins; *LDL*, low density lipoproteins; *VLDL*, very low density-lipoproteins

Data expressed as mean (standard error). ^a^P for quadratic trend; ^b^P for linear trend

Intra-treatments comparisons were made using general lineal model (GLM) with Bonferroni correction and age, sex and body weight at the beginning of the study as covariables. *p < 0.05; ^†^*p* < 0.01; ^≠^*p* < 0.001 versus its baseline; ^‡^*p* < 0.05; ^⁋^*p* < 0.001 versus 4 h. The inter-treatments comparisons were made using ANCOVA model with age, sex and body weight at the beginning of the study as covariables; ^¥^*p* < 0.05

#### Lipid and glycemic profile

The changes in the lipid and glycemic profiles at week 12 are shown in Table 5 and Supplementary item 4, respectively.

**Table 5:** Changes in glycaemic profile from baseline in abdominally obese subjects after supplementation with placebo or SIAP2 for 12 weeks

	SIAP2 (*n* = 57)	Placebo (*n* = 57)	SIAP2 vs. Placebo		Male				Female			
					SIAP2	Placebo	SIAP2 vs. Placebo		SIAP2	Placebo	SIAP2 vs. Placebo	
	MD (95% CI)	MD (95% CI)	MD	*p* value	MD (95% CI)	MD (95% CI)	MD	*p* value	MD (95% CI)	MD (95% CI)	MD	*p* value
*Glucose (mg/dL)*												
Δ Week 12-Baseline	− 0.335 (− 3.60; 2.93)	2.596 (− 0.60; 5.80)	− 2.93	0.207	− 1.388 (− 5.77; 3.00)	1.164 (− 3.01; 5.34)	− 2.552	0.405	1.285 (− 4.03; 6.60)	5.125 (− 0.34; 10.6)	− 3.840	0.317
*Insulin (mg/dL)*												
Δ Week 12-Baseline	− 5.585 (− 9.10; − 2.07)*	− 0.336 (− 3.78; 3.11)	− 5.249	0.037*	− 9.083 (− 14.4; 3.74)	− 0.413 (− 5.50; 4.67)	− 8.670	0.023*	0.233 (− 3.22; 3.67)	− 0.696 (− 4.24; 2.84)	0.919	0.711
*HOMA-IR*												
Δ Week 12-Baseline	− 1.355 (− 2.29; − 0.42)*	− 0.026 (− 0.93; 0.88)	− 1.329	0.047*	− 2.287 (− 3.71; − 0.86)*	− 0.073 (− 1.43; 1.28)	− 2.214	0.030*	0.184 (− 0.72; 1.09)	− 0.036 (− 0.94; 0.87)	0.220	0.732

Data expressed as mean (95% confidence interval, CI). HOMA calculated as (Glucose × Insulin)/405

*HOMA-IR* homeostatic model assessment

ANCOVA Model adjusted by sex, age, basal values, and physical activity at the beginning of the study. *p < 0.05

In the acute study, the TG level was increased 2 and 4 h (*p* < 0.05) after both treatments. At 4 h, SIAP2 consumption induced a significantly greater decrease in the TG concentration from the baseline (23.9 ± 7.03 mg/dL) compared with the placebo group (49.0 ± 9.52 mg/dL), which led to significant differences between the groups. At 4 h in the acute study, the reduction in TGs from the baseline detected in the SIAP2 group was double that found in the placebo group (Table 4).

The explanation of the other lipid and glycemic parameters are explained in Supplementary item 5.

### Gut microbiota

**Fig. 2:**
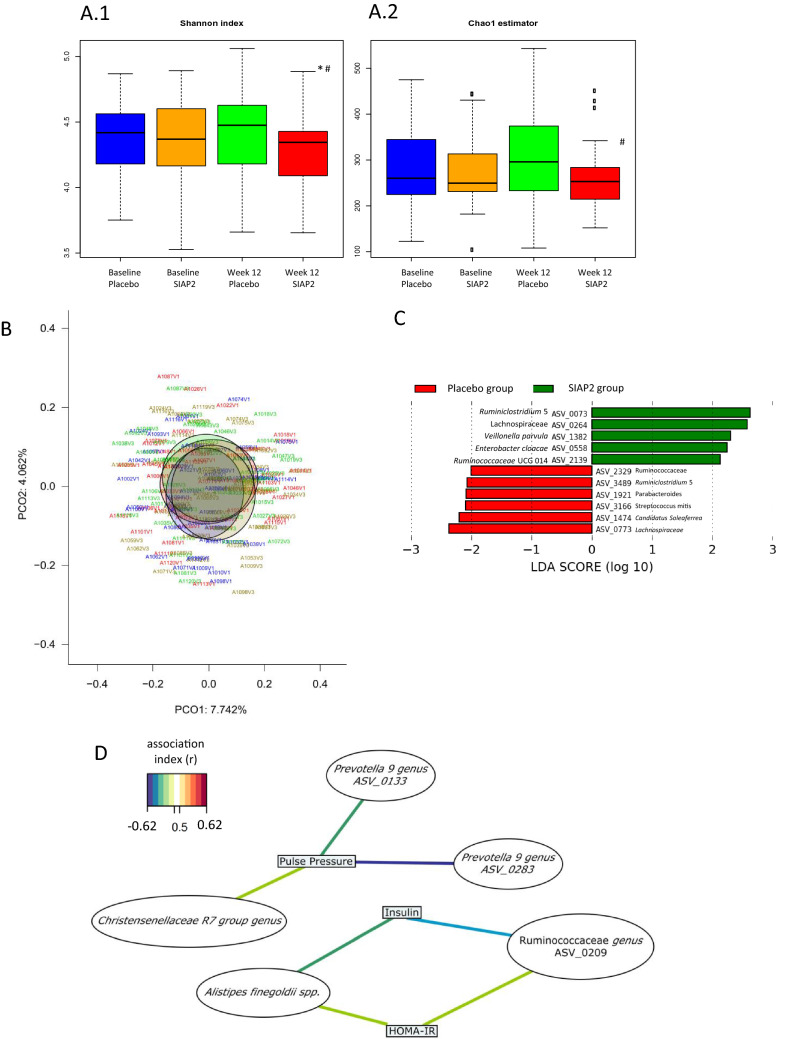
(**A**) Comparison of gut microbiota α-diversity indexes between both group of intervention (Placebo and SIAP2) at baseline and after 12 weeks of intervention: (**A.1**) diversity differences (Shannon index), (**A.2**) richness differences (Chao 1 index). *p* < 0.05 for intra-treatment comparisons. ^#^*p* < 0.02 for inter-treatment comparisons at 12 weeks of intervention (vs. Placebo); (**B**) Principal coordinates analysis (PCoA) based on Bray–Curtis dissimilarity index. (**C**) Linear discriminant analysis effect size (LEfSe) describing the differences between bacterial groups in abdominally obese subjects before (baseline) and after consuming SIAP2 for 12 weeks (LDA score > 2.0). (**D**) Relevant associations network, between clinical parameters that observed significant changes after 12 weeks of SIAP2 treatment and gut microbiota, at ASV level (*r* > 0.5) performed using a multivariant method (sparce partial least square). *Color lines show the correlation between ASVs and clinical variables. The positive and negative associations were related with line colors (association index)

At 12 weeks, the α-diversity, at the ASV level based on the Shannon index, as shown in Fig. 2A.1, and the richness based on the Chao1 estimator, as illustrated in Fig. 2A.2, showed that the gut microbiota of the SIAP2 group exhibited significantly less diversity than that of the placebo group (*p* = 0.0184). Moreover, the SIAP2 group presented significantly less diversity and richness at 12 weeks compared the levels observed at baseline (*p* = 0.04332 and *p* = 0.01142, respectively).

A β-diversity analysis was performed to identify the changes in the two microbiota structures produced by the interventions. This analysis showed non-significant changes in the gut microbiota at the ASV level between treatments (Adonis, *p* = 0.52; Fig. 2B).

Through LEfSE analysis, we identified a total of 63 ASV biomarkers that were significantly characteristic of each group (SIAP2 and placebo) with an LDA score > 2.0 at 12 weeks of intervention: 27 of these ASVs were enriched in the SIAP2 group, and 36 ASV biomarkers presented higher abundance in the placebo group (Supplementary Fig. S2). Moreover, Supplementary item 6 shows those biomarkers that presented higher discriminant power in each group with LDA scores > 2.5. The ASV biomarkers of the SIAP2 group with high LDA scores after 12 weeks of intervention mainly belonged to the families Ruminococcaceae, Lachnospiraceae, and Prevotellaceae and the genera *Prevotella*, *Ruminiclostridium*, *Lachnospiraceae* NK4A136, *Faecalibacterium*, *Roseburia*, *Blautia*, and *Holdemanella*. In particular, the genus *Lachnospiraceae* NK4A136 (LDA score = 2.7871, *p* = 0.0087) exhibited the highest discriminant power in the SIAP2 group. In contrast, at 12 weeks of intervention, the placebo group presented significantly higher abundance of a member of the family Ruminococcaceae (ASV_0209), *Coprococcus*, *Phascolarctobacterium faecium*, *Bacteroides eggerthii*, *Parabacteroides distasonis*, *Alistipes finegoldii* and *Bacteroides clarus*.

Based on the ASV biomarker abundance in the SIAP2 group after 12 weeks of intervention compared with the baseline, we identified a total of 11 ASV biomarkers that were significantly distinctive (LDA score > 2.0), as described in Fig. 2C: six ASV biomarkers at baseline and five ASV biomarkers after 12 weeks of intervention.

After 12 weeks of intervention, the SIAP2 group presented higher abundances of bacterial members of the families Lachnospiraceae, *Ruminiclostridium*, *Ruminococcaceae UCG-014*, *Enterobacter* and *Veillonella parvula*.

To further comprehend the role of the gut microbiota in the health status of the subjects, we performed an sPLS analysis using the clinical parameters that were significantly changed in the SIAP2 group after 12 weeks of intervention compared with the baseline and the ASV biomarkers of the SIAP2 and placebo groups (association index *r* > 0.5; Fig. 2D and Supplementary Fig. S3 and, Supplementary Item 7).

## Discussion

The analysis of the effects of sustained SIAP2 consumption on CMD risk factors, which could be partially due to changes in the gut microbiota composition, in subjects with abdominal obesity revealed a significant decrease in the PP in women in the SIAP2 group and significant decreases in insulin and HOMA-IR in the SIAP2 group, particularly in men, compared with the placebo group, at 12 weeks. Moreover, at 12 weeks, the increase in serum TG was reduced after SIAP2 consumption in the acute study compared with the placebo treatment. Additionally, the significant decreases in glycemic parameters, including insulin and HOMA-IR, observed after 12 weeks of SIAP2 consumption were related to higher abundances of *Alistipes finegoldii* and the family *Ruminococcaceae*. The significant decrease in the PP detected in women after 12 weeks of the SIAP2 intervention was related to higher abundances of *Prevotella 9-*ASV0283 and the *Christensenellaceae R7* group.

The average reduction in the PP of − 4.09 mmHg after 12 weeks of SIAP2 consumption, which was observed only in women, was higher than the range found in other studies after the consumption of a synbiotic (probiotics with fructooligosaccharides (FOS)) and alpha-tocopherol for 8 weeks [35]. A decrease in the PP can reduce the risk of cardiovascular disease. As observed in a prospective cohort study, an increase of 10 mmHg in the PP is related to increases of 13% and 20% in the rates of all-cause mortality and myocardial infarction [36], respectively.

SIAP2 treatment decreased the fasting insulin level by − 5.25 mg/dL and HOMA-IR, a surrogate marker of insulin resistance, by − 1.33 compared with the placebo group. The magnitude of the reduction in insulin was comparable to that obtained after 12 weeks of synbiotic consumption (inulin and *L. acidophilus*, *L. casei* and *B. bifdum*) [37]. Interestingly, similar results concerning HOMA-IR reduction by − 0.596 was observed in our previous study evaluating the effects of the postbiotic *Bifidobacterium animalis* subsp. *lactis* CECT 8145 consumption compared with its baseline, consumed in capsule form [38] for 12 weeks, which showed that the HOMA-IR reduction was maintained with a different dietary matrix.

In the present study, volunteers maintained their body weight throughout the intervention period, supporting the benefits of the enriched seafood sticks in a trade-off against the sugar, fat, and calorie counts [39, 40]. Moreover, the analysis of fat accumulation revealed no significant results after SIAP2 consumption, which differs from the effects related to the consumption of probiotics or postbiotics alone [41, 42], as observed with *Bifidobacterium animalis* subsp. *lactis* CECT 8145 consumption [38]. Thus, the consumption of probiotics or postbiotics with other bioactive compounds may reduce the effects on anthropometric parameters.

Our evaluation of the acute effects of SIAP2 after 12 weeks of sustained consumption compared with the placebo revealed a beneficial effect on the reduction in the total cholesterol/HDL cholesterol ratio and, the increase in the ApoA1/ApoB100 ratio after SIAP2 consumption from its baseline. In contrast, these results were not observed when we compare with its baseline in other acute study with the consumption of a synbiotic containing *Lactobacillus acidophilus, Bifidobacterium bifidum* and FOS [43]. Different results can be explained because the present acute study was performed after sustained consumption for 12 weeks, which optimizes the acute effects. Thus, the sustained consumption of SIAP2 can exert beneficial postprandial effects on the lipid profile.

Most remarkably, in the acute study, the expected increase in the serum TG concentration was reduced 4-h after SIAP2 consumption compared with the placebo. The TG reduction can be explained by the content of omega-3 present in the SIAP2 product. This explanation is supported by a meta-analysis of RCT with T2D adults, which found that the consumption of omega-3 (average of 1.12–2.6 g of EPA + 0.80–1.60 g of DHA per day), induces a small reduction in postprandial hypertriglyceridemia (mean of 0.39, 95% CI − 0.55 to − 0.24; *p* ≤ 0.001 mmol/L [44]; equivalent to 18 mg/dL). The doses consumed in the RCTs with T2D adults included in the meta-analyses were higher than those used in the present study per day (370 mg of EPA + DHA).

Thus, the acute effects of SIAP2 on serum TG reduction, half of concentration compared with the placebo, detected with the inclusion of lower doses (370 mg) of SIAP2 containing EPA + DHA were similar to those found in other RCTs evaluating the effects on TGs after the consumption of EPA + DHA alone [44].

The mechanisms of action underlying the effects of SIAP2 consumption can be related to the gut microbiota. In the present work, a decrease in α-diversity after SIAP2 consumption was observed, which suggested that SIAP2 consumption favored the overgrowth of specific bacteria associated with a great health status [45]; thus, the microbiota were enriched in fiber-degrader microbiota such as *Prevotella* and *Ruminiclostridium* and in butyrate-producer bacteria such as the *Lachnospiraceae* NK4A136 group, *Faecalibacterium, Roseburia* and *Holdemanella *[46, 47]*. Holdemanella biformis* antitumoral activity through the production of short-chain fatty acids (SCFAs) [48]. Moreover, the *Prevotella* genus has been described as a propionate producer, and its SCFAs play an important role in reducing serum cholesterol and hepatic lipogenesis [49]. We also found that the genera *Prevotella 9-*ASV0283 and family Christensenellaceae were negatively correlated with the PP. Thus, the enrichment of *Prevotella* and other beneficial bacteria after the consumption of SIAP2 for 12 weeks could be at least partially responsible for the decrease in the PP detected in women. Other authors have also stated that the family Christensenellaceae is negatively correlated with BP [50] and positively associated with low CMD risk [50]. In Chinese population, Christensenellaceae is positively correlated with fecal branched-chain fatty acids (BCFAs) [51]. These BCFAs are able to dehydroxylate or deconjugate bile acids such as lithocholic acids (LCAs) and hyodeoxycholic acid (HDCA). Thus, LCA and HDCA in the gut microbiota can influence the bile acid-activated farnesoid X receptor signaling, which regulates host glucose homeostasis by improving insulin sensitivity and lipid metabolism [52]. After analyzing the gut microbiota differences between men and women after the consumption of SIAP2 for 12 weeks, we observed that gut microbes did not match the gut microbiota biomarkers identified with changes in the PP. In consequence, more studies are needed to determine the reason for these differences and to confirm the existence of a connection between both microbial changes observed in the present work (the gut microbiota differences between men and women and the gut microbiota biomarkers identified with changes in the PP).

Most remarkably, after the consumption of SIAP2 for 12 weeks, a negative association for a genus of the family Ruminococcaceae and *Alistipes finegoldii* with serum insulin and HOMA-IR was observed. In this sense, a published review revealed that subjects without diabetes present higher abundance of the family Ruminococcaceae than subjects with diabetes, suggesting a protective effect on increase serum glucose [53].

The present study has several strengths. To the best of our knowledge, the present work has demonstrated novel and beneficial effects of a combination of postbiotic and bioactive compounds related to CMD risk factors whereas the diet control during 12 weeks of intervention showed no differences between two groups, supporting the possible mechanisms of action underlying the reduction of insulin due to changes in the gut microbiota.

However, more studies are needed to elucidate the mechanisms of action through which synbiotic or postbiotics with bioactive compounds influence the gut microbiota.

Additionally, the present study has some limitations, such as the inability to assess potential interactions between the intervention product and other dietary components that may affect the results. Furthermore, our subjects had abdominal obesity, which may hamper the extrapolation of the results to the general population since long-term effects of SIAP2 consumption remain unknown. Moreover, compliance with the intervention was not directly evaluated through the quantification of heat-inactivated *B. animalis* subsp. *lactis* CECT 8145 in fecal samples; instead, product compliance was indirectly assessed by counting the number of empty packages based on the fact thatf the consumption of almost 2 additional servings of fish per week might ameliorate the dietary habits of the subjects and in turns their health. Finally, the authors can only speculate on the role of the gut microbiota effects, as they do not measure any of the products of the metabolism, such as SCFA.

## Conclusion

In summary, in subjects with abdominal obesity, sustained consumption of seafood sticks enriched with postbiotic and bioactive compounds induces reductions in the insulin concentrations and HOMA-IR compared with the levels obtained with conventional seafood sticks, and these effects potentially protect against the development of T2D. Furthermore, the consumption of the enriched seafood sticks also significantly reduces the PP in women that can prevent the cardiovascular disease. These effects are accompanied by partial changes in the gut microbiota composition. Moreover, the seafood sticks enriched with postbiotic and bioactive compounds reduce the postprandial atherogenic TG concentrations compared with the levels obtained with conventional seafood sticks. The gut microbiota changes were related to reductions in serum insulin (due to increases abundance of *Alistipes finegoldii* and Ruminococcaceae genus) and the PP (due to increases in *Prevotella 9-*ASV0283 *and* the *Christensenellaceae R7* group). Our results support the use of seafood sticks enriched with postbiotic and bioactive compounds as a complementary strategy in the management of CMD risk factors.

## Supplementary Information

Below is the link to the electronic supplementary material.

Supplementary file1 (DOCX 45 KB)

Supplementary file2 (PPTX 5268 KB)
