# Supplementary material for: Effects of enriched seafood sticks (heat-inactivated B. animalis subsp. lactis CECT 8145, inulin, omega-3) on cardiometabolic risk factors and gut microbiota in abdominally obese subjects: randomized controlled trial
Source: Eur J Nutr. 2022 May 28;61(7):3597–611. doi: 10.1007/s00394-022-02904-0 (PMC9464132; doi:10.1007/s00394-022-02904-0)
Supplement: Supplementary file 1 — Supplementary file1 (DOCX 45 KB) [file 394_2022_2904_MOESM1_ESM.docx]

SUPPLEMENTARY INFORMATION (8 Items)

**Supplementary item 1. Baseline characteristics of the participants according to the intervention group**

|  | | | |
| --- | --- | --- | --- |
|  | **SIAP2**  **(n = 60)** | **Placebo**  **(n = 60)** | *P*-value |
| Age. *y* | 51.0 ± 8.8 | 50.9 ± 11.3 | 0.964 |
| Female. *%* | 42.4 | 40.0 | 0.793 |
| Smoking habits. *%* |  |  |  |
| Never | 25.4 | 39.7 | 0.194 |
| Smoker | 27.1 | 17.2 | 0.239 |
| Ex smoker | 47.5 | 43.1 | 0.680 |
| Systolic blood pressure, *mm Hg* | 134 ± 18 | 131 ± 16 | 0.422 |
| Diastolic blood pressure, *mm Hg* | 85 ± 11 | 82 ± 10 | 0.197 |
| Pulse pressure, *mm Hg* | 49 ± 10 | 49 ± 11 | 0.933 |
| Body mass index, *kg/m^2^* | 32.0 ± 3.5 | 31.1 ± 3.6 | 0.156 |
| Waist circumference, *cm* | 107.3 ± 10.0 | 105.4 ± 10.5 | 0.317 |
| Waist/height | 0.65 ± 0.06 | 0.64 ± 0.06 | 0.215 |
| Conicity index | 1.35 ± 0.09 | 1.34 ± 0.07 | 0.444 |
| Fat mass,*%* | 35.2 ± 8.6 | 35.3 ± 8.7 | 0.964 |
| Fat mass, *kg* | 31.5 ± 9.1 | 30.8 ± 9.2 | 0.674 |
| Lean mass, *kg* | 58.9 ± 14.9 | 56.8 ±12.9 | 0.425 |
| Muscle mass, *kg* | 56.0 ± 14.2 | 54.0 ± 12.3 | 0.422 |
| Bone mass, *kg* | 2.94 ± 0.70 | 2.84 ± 0.60 | 0.413 |
| Total water, *%* | 46.6 ± 5.6 | 46.8 ± 7.2 | 0.832 |
| Total water, *kg* | 42.6 ± 10.7 | 41.1 ± 10.6 | 0.459 |
| Physical activity, *AU** | 9.0 (4.0-15) | 11 (4.0-18) | 0.531 |
| Abbreviations: AU, arbitrary units: 0-1, inactive; 2-3, very low activity; 4–5, low activity; 6–11 moderately active; > or ≥ 12, very active.  Data expressed as mean ± standard deviation, or percentages. *median (25^th^ – 75^th^ percentiles).  *P*-value for gender, physical activity and smoking habits was calculated by Chi-Square test. *P*-value for all other variables was calculated by Student’s t-test and Mann–Whitney U test. **P*<0.05. | | | |

**Supplementary item 2. Dietary composition before and after treatment interventions.**

|  | | | | | | | |
| --- | --- | --- | --- | --- | --- | --- | --- |
|  | **SIAP2**  (n=57) | | | **Placebo**  (n=57) | | | *P*-value between groups |
|  | Before  (Week 0) | After  (Week 12) | *P-*value (within group) | Before  (Week 0) | After  (Week 12) | *P*-value (within group) |  |
| Energy, Kcal | 2040 ± 508 | 1929 ± 557 | 0.139 | 2027 ± 683 | 1990 ± 719 | 0.603 | 0.472 |
| Carbohydrates, g | 177 ± 55 | 165 ± 60 | 0.106 | 182 ± 65 | 168 ± 72 | 0.043 | 0.859 |
| Carbohydrates, % | 35.3 ± 6.7 | 34.7 ± 7.1 | 0.585 | 37.0 ± 5.8 | 34.5 ± 7.1 | 0.006 | 0.189 |
| Protein, g | 93.7 ± 23.5 | 83.5 ± 25.8 | 0.053 | 86.1 ± 26.2 | 86.5 ± 33.1 | 0.895 | 0.101 |
| Protein, % | 18.7 ± 5.2 | 17.9 ± 3.7 | 0.139 | 17.6 ± 3.2 | 17.9 ± 4.0 | 0.494 | 0.128 |
| Lipid, g | 96.5 ± 28.8 | 92.4 ± 31.7 | 0.446 | 96.4 ± 39.3 | 97.5 ± 42.8 | 0.810 | 0.460 |
| Lipid, % | 42.0 ± 5.8 | 42.6 ± 6.7 | 0.595 | 41.3 ± 5.8 | 43.1 ± 8.4 | 0.100 | 0.486 |
| SFA, g | 29.3 ± 11.5 | 27.5 ± 11.9 | 0.381 | 29.4 ± 14.3 | 28.4 ± 14.2 | 0.578 | 0.785 |
| SFA, % | 12.4 ± 2.9 | 12.4 ± 2.7 | 0.970 | 12.5 ± 2.8 | 12.4 ± 3.4 | 0.833 | 0.904 |
| PUFA, g | 29.3 ± 11.5 | 27.5 ± 11.9 | 0.381 | 29.4 ± 14.3 | 28.4 ± 14.2 | 0.578 | 0.233 |
| PUFA, % | 6.8 ± 2.1 | 7.1 ± 2.5 | 0.538 | 6.5 ± 2.0 | 7.4 ± 2.3 | 0.004 | 0.179 |
| MUFA, g | 43.2 ± 12.6 | 41.9 ± 14.4 | 0.602 | 43.9 ± 17.1 | 44.2 ± 18.8 | 0.883 | 0.618 |
| MUFA, % | 19.1 ± 3.4 | 19.7 ± 3.8 | 0.398 | 19.0 ± 3.1 | 19.7 ± 4.5 | 0.243 | 0.904 |
| Fiber, g | 18.4 ± 6.4 | 17.4 ± 8.7 | 0.259 | 19.3 ± 7.3 | 16.6 ± 6.1 | 0.004 | 0.203 |
| Alcohol, g | 4.26 (0.00-19.7) | 7.40 (2.7-18.1) | 0.320 | 5.87 (0.26-19.6) | 7.55 (0.00-16.8) | 0.450 | 0.747 |
| Sodium, g** | 2.71 ± 0.80 | 2.39 ± 1.07 | 0.076 | 2.37 ± 1.03 | 2.34 ± 1.17 | 0.857 | 0.124 |
| Abbreviations: CHO, carbohydrates; SFA, saturated fatty acids; MUFA, monounsaturated fatty acids; PUFA, polyunsaturated fatty acids.  Data expressed as Mean ± standard deviation: a median (25th-75thpercentile).  Intra-treatment comparisons by Student’s t test and Wilcoxon test for related samples.  * P value for differences among treatments. ANOVA and Mann–Whitney tests.  **Do not include the added salt at foods | | | | | | | |

|  | | | | | | | | | | | | |
| --- | --- | --- | --- | --- | --- | --- | --- | --- | --- | --- | --- | --- |
|  | | | | | **Male** | | | | **Female** | | | |
|  | SIAP2  (n=57) | Placebo  (n=57) | SIAP2  vs.  Placebo | | SIAP2 | Placebo | SIAP2  vs.  Placebo | | SIAP2 | Placebo | SIAP2  vs.  Placebo | |
|  | MD  (95% CI) | MD  (95% CI) | MD | *P*  value | MD  (95% CI) | MD  (95% CI) | MD | *P*  value | MD  (95% CI) | MD  (95% CI) | MD | *P*  value |
| **Body weight, *kg*** | | | | | | | | | | | | |
| Δ Week 6-Baseline | -0.560  (-1.03; -0.09)* | 0.179  (-0.29;0.65) | -0.740 | 0.030* | -0.460  (-1.05;0.13) | 0.252  (-0.33;0.83) | -0.713 | 0.094 | -0.670  (-1.49;0.15) | 0.031  (-0.81;0.87) | -0.701 | 0.237 |
| Δ Week 12-Baseline | -0.258  (-1.10;0.58) | -0.037  (-0.87;0.79) | -0.220 | 0.220 | -0.475  (-1.72;0.77) | 0.058  (-1.15;1.27) | -0.533 | 0.544 | 0.116  (-0.86;1.09) | -0.239  (-1.24;0.76) | 0.354 | 0.610 |
| **BMI, *kg/m^2^*** | | | | | | | | | | | | |
| Δ Week 6-Baseline | -0.007  (-0.19; 0.18) | 0.040  (-0.15;0.23) | -0.047 | 0.732 | 0.046  (-0.24;0.34) | 0.048  (-0.24;0.34) | -0.002 | 0.994 | -0.069  (-0.27;0.13) | 0.011  (-0.19;0.21) | -0.080 | 0.575 |
| Δ Week 12-Baseline | -0.681  (-1.60;0.24) | -0.005  (-0.93;0.92) | -0.676 | 0.310 | -1.110  (-2.59;0.37) | 0.003  (-1.45;1.46) | -1.113 | 0.290 | 0.056  (-0.32;0.43) | -0.059  (-0.44;0.32) | 0.116 | 0.665 |
| **WC, *cm*** | | | | | | | | | | | | |
| Δ Week 6-Baseline | -0.637  (-1.18; -0.10)* | -0.786  (-1.34; -0.23)* | 0.149 | 0.704 | -0.660  (-1.38;0.06) | -1.091  (-1.72; -0.28)* | 0.341 | 0.508 | -0.554  (-1.41;0.30) | -0.488  (-1.39;0.41) | -0.066 | 0.917 |
| Δ Week 12-Baseline | -0.678  (-1.57;0.21) | -0.602  (-1.49;0.29) | -0.075 | 0.906 | -0.824  (-1.97;0.32) | -0.447  (-1.56;0.66) | -0.377 | 0.639 | -0.437  (-1.96;1.08) | -0.904  (-2.51;0.70) | 0.467 | 0.674 |
| **WC /Height** | | | | | | | | | | | | |
| Δ Week 6-Baseline | -0.004  (-0.007; -0.000)* | -0.004  (-0.008; -0.001)* | 0.001 | 0.720 | -0.004  (-0.008; -0.000)* | -0.006  (-0.01; -0.002)* | 0.002 | 0.474 | -0.003  (-0.01;0.003) | -0.002  (-0.008; 0.004) | -0.002 | 0.873 |
| Δ Week 12-Baseline | -0.004  (-0.01;0.001) | -0.004  (-0.009 ;0.002) | -0.001 | 0.891 | -0.005  (-0.01;0.002) | -0.002  (-0.009;0.004) | -0.003 | 0.568 | -0.003  (-0.01;0.007) | -0.005  (-0.01;0.005) | 0.002 | 0.804 |
| **Conicity index** | | | | | | | | | | | | |
| Δ Week 6-Baseline | -0.005  (-0.01;0.003) | -0.009  (-0.02;0.000) | 0.004 | 0.540 | -0.006  (-0.01;0.003) | -0.015  (-0.02; -0.006)* | 0.009 | 0.126 | -0.004  (-0.02;0.01) | 0.001  (-0.02;0.02) | -0.004 | 0.728 |
| Δ Week 12-Baseline | -0.007  (-0.02;0.002) | -0.008  (-0.02;0.002) | 0.000 | 0.954 | -0.007  (-0.02;0.005) | -0.006  (-0.02;0.005) | -0.001 | 0.920 | -0.005  (-0.02;0.01) | -0.013  (-0.03;0.003) | 0.008 | 0.504 |
| Abbreviations: BMI, body mass index (weight/height^2^ [in meters]); WC, waist circumference; Waist/Height, waist (cm)/ height (cm) ratio; MD, mean difference.  Data expressed as mean (95% Confidence Interval, CI).  ANCOVA Model adjusted by sex, age, basal values, and physical activity at the beginning of the study. *P<0.05. | | | | | | | | | | | | |

**Supplementary item 3. Changes in anthropometrics and adiposity parameters from baseline in abdominally obese subjects after supplementation with placebo or SIAP2 for 6 and 12 weeks.**

|  | | | | | | | | | | | | |
| --- | --- | --- | --- | --- | --- | --- | --- | --- | --- | --- | --- | --- |
|  | | | | | **Male** | | | | **Female** | | | |
|  | SIAP2  (n=57) | Placebo  (n=57) | SIAP2 vs. Placebo | | SIAP2 | Placebo | SIAP2 vs. Placebo | | SIAP2 | Placebo | SIAP2 vs. Placebo | |
|  | MD  (95% CI) | MD  (95% CI) | MD | *P*  value | MD  (95% CI) | MD  (95% CI) | MD | *P*  value | MD  (95% CI) | MD  (95% CI) | MD | *P*  value |
| **Total cholesterol, *mg/dL*** | | | | | | | | | | | | |
| Δ Week 12-Baseline | 1.013  (-4.55;6.58) | -3.312  (-8.71;2.08) | 4.326 | 0.272 | -0.242  (-8.24;7.76) | -3.757  (-11.2;3.72) | 3.514 | 0.524 | 2.748  (-4.81;10.3) | -2.401  (-10.2;5.37) | 5.149 | 0.345 |
| **HDL cholesterol, *mg/dL*** | | | | | | | | | | | | |
| Δ Week 12-Baseline | -0.490  (-2.17;1.19) | -1.610  (-3.26; 0.04) | 1.120 | 0.349 | -0.344  (-2.33;1.64) | -2.125  (-4.01; -0.24)* | 1.781 | 0.200 | -0.557  (-3.66;2.54) | -0.856  (-4.04;2.33) | 0.299 | 0.893 |
| **LDL cholesterol, *mg/dL*** | | | | | | | | | | | | |
| Δ Week 12-Baseline | 0.971  (-4.48;6.42) | -0.773  (-6.05;4.51) | 1.744 | 0.650 | -0.459  (-8.06;7.15) | -1.474  (-8.58;5.64) | 1.015 | 0.846 | 2.488  (-5.10;10.1) | 1.096  (-6.70;8.89) | 1.392 | 0.798 |
| **VLDL cholesterol, *mg/dL*** | | | | | | | | | | | | |
| Δ Week 12-Baseline | 0.918  (-1.56;3.39) | -0.942  (-3.37;1.48) | 1.860 | 0.290 | 1.152  (-2.48;4.78) | -0.138  (-3.59;3.32) | 1.290 | 0.610 | 0.817  (-2.19;3.83) | -2.641  (-5.74;0.46) | 3.458 | 0.116 |
| **Total cholesterol/HDL cholesterol** | | | | | | | | | | | | |
| Δ Week 12-Baseline | 0.102  (-0.02;0.22) | 0.061  (-0.06;0.18) | 0.042 | 0.630 | 0.094  (-0.09;0.28) | 0.091  (-0.08;0.26) | 0.003 | 0.980 | 0.099  (-0.04;0.23) | 0.023  (-0.12;0.16) | 0.076 | 0.431 |
| **LDL cholesterol/ HDL cholesterol** | | | | | | | | | | | | |
| Δ Week 12-Baseline | 0.066  (-0.04;0.18) | 0.053  (-0.05;0.16) | 0.013 | 0.871 | -0.459  (-8.06;7.15) | -1.474  (-8.58;5.64) | 1.015 | 0.846 | 2.488  (-5.10;10.1) | 1.096  (-6.70;8.89) | 1.392 | 0.798 |
| **Triglycerides, *mg/dL*** | | | | | | | | | | | | |
| Δ Week 12-Baseline | 4.465  (-7.80;16.7) | -4.786  (-16.8;7.23) | 9.251 | 0.289 | 5.270  (-12.8;23.3) | -0.745  (-17.9;16.4) | 6.015 | 0.632 | 4.543  (-10.3;19.4) | -13.351  (-28.6;1.91) | 17.893 | 0.100 |
| **NEFA, *mmol/L*** | | | | | | | | | | | | |
| Δ Week 12-Baseline | 0.061  (-0.04;0.16) | 0.083  (-0.01;0.18) | -0.022 | 0.752 | 0.069  (-0.07;0.21) | 0.106  (-0.03;0.24) | -0.037 | 0.706 | 0.052  (-0.09;0.19) | 0.039  (-0.10;0.18) | 0.013 | 0.898 |
| **Apo A1, *mg/dL*** | | | | | | | | | | | | |
| Δ Week 12-Baseline | -2.143  (-5.64;1.35) | -1.670  (-5.16;1.83) | -0.473 | 0.850 | -2.201  (-6.3;1.91) | -4.070  (-8.05; -0.09)* | 1.869 | 0.517 | -1.274  (-7.34;4.80) | 1.835  (-4.29;8.26) | -3.109 | 0.482 |
| **Apo B100, *mg/dL*** | | | | | | | | | | | | |
| Δ Week 12-Baseline | 0.404  (-3.91;4.71) | -3.763  (-8.03; 0.50) | 4.168 | 0.177 | 1.019  (-5.59;7.63) | -3.727  (-10.1;2.67) | 4.746 | 0.308 | -1,015  (-5.39;3.36) | -3.318  (-7.82;1.18) | 2.303 | 0.465 |
| **Apo A1/Apo B100 ratio** | | | | | | | | | | | | |
| Δ Week 12-Baseline | -0.005  (-0.06;0.05) | 0.022  (-0.03;0.08) | -0.028 | 0.492 | -0.003  (-0.07;0.07) | -0.008  (-0.08;0.06) | 0.006 | 0.370 | 0.006  (-0.08;0.09) | 0.061  (-0.03;0.15) | -0.056 | 0.370 |
| Abbreviations: Apo, Apolipoprotein; HDL, high-density lipoproteins; LDL, low density lipoproteins; VLDL, very low-density lipoproteins, NEFA, non-esterified fatty acids, MD, mean difference.  Data expressed as mean (95% Confidence Interval, CI).  ANCOVA Model adjusted by sex, age, basal values, and physical activity at the beginning of the study. *P*<0.05. | | | | | | | | | | | | |

**Supplementary item 4. Changes in lipid profile from baseline in abdominally obese subjects after supplementation with placebo or SIAP2 for 12 weeks**

**Supplementary item 5. Lipid and glycemic profile**

After 12 weeks of intervention, insulin and HOMA-IR were reduced in the SIAP2 group, and the decreases reached significance compared with the changes after placebo treatment (*p* < 0.005). The gender-based analysis of the data revealed that the decreases in insulin and HOMA-IR were significant only in men (*p* < 0.05), as shown in ***Table 5***.

In the acute study, a similar glucose and insulin pattern was observed after both treatments, as shown in ***Table 4***. The glucose was increased at 2 h, and this increase was significant in the SIAP2 group (*p* < 0.01), whereas a borderline decrease (*p* < 0.01) was observed 4 h after both treatments (***Table 4***). Similar increases in insulin (*p* < 0.01) were detected 2 h after both interventions, and no differences were observed between the treatments.

At 12 weeks, no intra- or intertreatment differences in the lipid or Apo profiles were observed. The analysis of the data by gender revealed decrease in HDL cholesterol and Apo A-1 only in men after the placebo treatment (p < 0.05), and no changes in the other variables were detected, as described in **Supplementary item 4**.

The results of the acute study of the lipid profile showed that the total cholesterol level was decreased at 2 h and that this decrease reached significance only after SIAP2 consumption (*p* < 0.01), as shown in ***Table 4***. At 2 and 4 h after both treatments, the levels of HDL and LDL cholesterol were decreased and that of VLDL cholesterol was increased (*p* < 0.01). The total cholesterol/HDL cholesterol ratio was increased 2 h after both treatments (*p* < 0.05), but a decrease at 4 h was only observed after SIAP2 consumption (*p* < 0.05). The LDL/HDL cholesterol ratio was similarly increased at 2 h (*p* < 0.01) and decreased at 4 h (*p* < 0.001) after both treatments. No intertreatment differences in the abovementioned variables were observed (***Table 4***).

In the acute study, NEFA was decreased at 2 h only after the placebo treatment (*p* < 0.05), but no intertreatment differences were detected.

ApoA-1 was similarly decreased at 2 h after both treatments, and the decrease reached significance after SIAP2 consumption (*p* < 0.01). Apo B-100 was decreased at 2 h and 4 h after both treatments (*p* < 0.05). The ApoA1/ApoB100 ratio was increased at 2 h (*p* < 0.05) and 4 h after both treatments, but a significant increase at 4 h was only observed in the SIAP2 group (*p* < 0.01). No differences between the treatments were observed (***Table 4***).

|  | | | |
| --- | --- | --- | --- |
| **ASV** | **LDA-SCORE**  **(log10)** | ***p-value*** | **Taxonomy** |
| **Placebo** | | | |
| ASV_0023 | 2.841 | 0.0176 | Bacteria;p__Firmicutes;c__Negativicutes;o__Selenomonadales;f__Acidaminococcaceae;g__Phascolarctobacterium;s__faecium |
| ASV_0149 | 2.746 | 0.0173 | Bacteria;p__Bacteroidetes;c__Bacteroidia;o__Bacteroidales;f__Bacteroidaceae;g__Bacteroides;s__eggerthii |
| ASV_0209 | 2.715 | 0.0385 | Bacteria;p__Firmicutes;c__Clostridia;o__Clostridiales;f__Ruminococcaceae;g__NA;s__NA |
| ASV_0224 | 2.636 | 0.0327 | Bacteria;p__Bacteroidetes;c__Bacteroidia;o__Bacteroidales;f__Tannerellaceae;g__Parabacteroides;s__distasonis |
| ASV_0143 | 2.616 | 0.0232 | Bacteria;p__Firmicutes;c__Clostridia;o__Clostridiales;f__Lachnospiraceae;g__Coprococcus_2;s__NA |
| ASV_0185 | 2.577 | 0.0223 | Bacteria;p__Bacteroidetes;c__Bacteroidia;o__Bacteroidales;f__Rikenellaceae;g__Alistipes;s__[finegoldii] |
| ASV_0320 | 2.522 | 0.0174 | Bacteria;p__Bacteroidetes;c__Bacteroidia;o__Bacteroidales;f__Bacteroidaceae;g__Bacteroides;s__clarus |
| **SIAP2** | | | |
| ASV_0116 | 2.787 | 0.0087 | Bacteria;p__Firmicutes;c__Clostridia;o__Clostridiales;f__Lachnospiraceae;g__Lachnospiraceae_NK4A136_group;s__NA |
| ASV_0084 | 2.767 | 0.0097 | Bacteria;p__Firmicutes;c__Clostridia;o__Clostridiales;f__Ruminococcaceae;g__NA;s__NA |
| ASV_0003 | 2.737 | 0.0143 | Bacteria;p__Firmicutes;c__Clostridia;o__Clostridiales;f__Ruminococcaceae;g__Faecalibacterium;s__prausnitzii |
| ASV_0051 | 2.730 | 0.0318 | Bacteria;p__Bacteroidetes;c__Bacteroidia;o__Bacteroidales;f__Prevotellaceae;g__Prevotella_9;s__NA |
| ASV_0040 | 2.700 | 0.0342 | Bacteria;p__Firmicutes;c__Clostridia;o__Clostridiales;f__Lachnospiraceae;g__NA;s__NA |
| ASV_0053 | 2.697 | 0.0202 | Bacteria;p__Bacteroidetes;c__Bacteroidia;o__Bacteroidales;f__Prevotellaceae;g__Prevotella_9;s__NA |
| ASV_0001 | 2.659 | 0.0127 | Bacteria;p__Firmicutes;c__Clostridia;o__Clostridiales;f__Ruminococcaceae;g__Faecalibacterium;s__prausnitzii |
| ASV_0033 | 2.640 | 0.0449 | Bacteria;p__Firmicutes;c__Clostridia;o__Clostridiales;f__Lachnospiraceae;g__Roseburia;s__inulinivorans |
| ASV_0063 | 2.610 | 0.0046 | Bacteria;p__Firmicutes;c__Clostridia;o__Clostridiales;f__Lachnospiraceae;g__Blautia;s__[obeum] |
| ASV_0219 | 2.602 | 0.0051 | Bacteria;p__Firmicutes;c__Erysipelotrichia;o__Erysipelotrichales;f__Erysipelotrichaceae;g__Holdemanella;s__[biformis DSM 3989] |
| ASV_0056 | 2.573 | 0.0464 | Bacteria;p__Bacteroidetes;c__Bacteroidia;o__Bacteroidales;f__Prevotellaceae;g__Prevotella_9;s__NA |
| ASV_0383 | 2.541 | 0.0419 | Bacteria;p__Firmicutes;c__Clostridia;o__Clostridiales;f__Ruminococcaceae;g__Ruminiclostridium_6;s__NA |
| **P* value for differences in ASV abundance among treatments (Placebo vs SIAP2). *P* value ≤ 0.05 | | | |

**Supplementary item 6. Significant differences between two groups (SIAP2 and placebo) in the relative abundance of ASVs, that present a LDA score >2.5 at 12 weeks of intervention.**

**Supplementary item 7. Further comprehension of the role of the gut microbiota in the health status of the subjects.**

The variables insulin and HOMA-IR showed a negative association with a bacterial member of Ruminococcaceae and *Alistipes finegoldii (****Figure 2D***), and these two variables are related and thus clustered together, as demonstrated by a similar association profile (***Supplementary Figure S3*)*.*** However, the PP showed a different profile with negative associations with *Prevotella 9* (ASVs: 0133 and 0283) and the *Christensenellaceae R7* group, as described in ***Figure 2D***.

Due to the differential responses between gender in the SIAP2 group, we performed an additional gut microbiota analysis to identify those bacterial microbes that were showed significant differences between genders after 12 weeks of SIAP2 treatment (***Supplementary Figure S4***): (1) A ß-diversity analysis was performed to identify changes in the microbiota structure in both genders, at baseline (***Supplementary Figure S4.A***) and after 12 weeks of the SIAP2 (***Supplementary Figure S4.B***) and placebo treatments (***Supplementary Figure S4.C***). This analysis only showed significant changes between genders after 12 weeks of SIAP2 treatment (Adonis, *p* = 0.016; ***Supplementary Figure S4.B***); (2) using LEfSE analysis identified a total of 34 ASV biomarkers that showed significant differences between men and women after 12 weeks of the SIAP2 treatment with an LDA score > 2.5: 14 ASVs were enriched in men, and 20 ASV biomarkers presented higher abundance in women. The ASV biomarkers enriched in men after 12 weeks of the SIAP2 treatment with an LDA score > 2.5 mainly belonged to the families Bacteroidaceae, Christensenellaceae, Family XIII of the order Clostridiales, Lachnospiraceae, Ruminococcaceae and Akkermansiaceae, and particularly to the genera *Bacteroides* (ASV_0025), *Butyricicoccus*, *Ruminococcus* and *Akkermansia* (***Supplementary Figure S4D***). In contrast, after 12 weeks of the SIAP2 treatment, women presented significantly high abundance of the families Bacteroidaceae, Lachnospiraceae and Ruminococcaceae and the genera *Intestinimonas,* *Bacteroides* (ASV_0158) and *Ruminiclostridium* 6 (***Supplementary Figure S4D).***

**Supplementary Figure Legends.**

**Supplementary Figure S1**. The design of the randomized controlled trial.

**Supplementary Figure S2**. The Linear discriminant analysis Effect Size (LEfSe). A) Describing the greatest differences between bacterial groups in abdominally obese subjects after consuming SIAP2 or placebo for 12 weeks (LDA score > 2.0).

**Supplementary Figure S3.** Heatmap of the associations between ASV biomarkers of SIAP2 and placebo groups, at 12 weeks of intervention, and differential clinical parameters between SIAP2, at 12 weeks of intervention, compared to baseline and those ASV biomarkers that were also changed after SIAP2 treatment.

**Supplementary Figure S4.** (**A)** ß-diversity analysis to identify changes of microbiota structure in both genders, at baseline. **(B)** ß-diversity analysis to identify changes of microbiota structure in both genders, after 12 weeks of SIAP2 intervention. **(C)** ß -diversity analysis to identify changes of microbiota structure in both genders, after placebo treatment for 12 weeks. (D) Linear discriminant analysis Effect Size (LEfSe) describing the differences between bacterial groups in men and women after 12 weeks of SIAP2 intervention (LDA score > 2.5).
