# Supplementary material for: Effects of enriched seafood sticks (heat-inactivated B. animalis subsp. lactis CECT 8145, inulin, omega-3) on cardiometabolic risk factors and gut microbiota in abdominally obese subjects: randomized controlled trial
Source: Eur J Nutr. 2022 May 28;61(7):3597–611. doi: 10.1007/s00394-022-02904-0 (PMC9464132; doi:10.1007/s00394-022-02904-0)
Supplement: Supplementary file 2 — Supplementary file2 (PPTX 5268 KB) [file 394_2022_2904_MOESM2_ESM.pptx]

## Slide 1
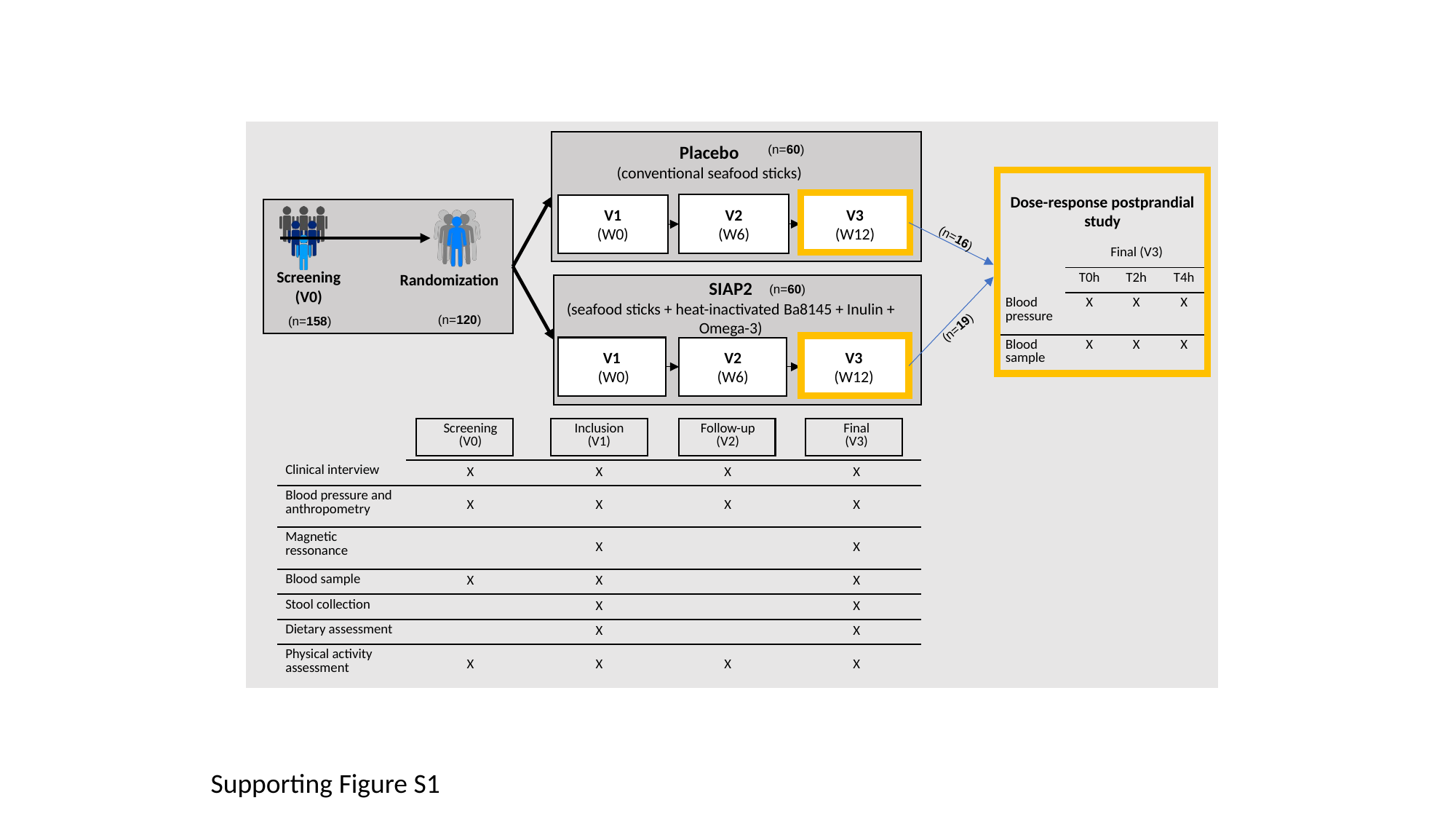

Placebo
(conventional seafood sticks)
(n=60)
Dose-response postprandial study
V2
(W6)
V3
(W12)
V1
(W0)
(n=16)
| | Final (V3) | | |
| --- | --- | --- | --- |
| | T0h | T2h | T4h |
| Blood pressure | X | X | X |
| Blood sample | X | X | X |
Screening (V0)
Randomization
SIAP2
(seafood sticks + heat-inactivated Ba8145 + Inulin + Omega-3)
(n=60)
(n=120)
(n=158)
(n=19)
V1
 (W0)
V3
(W12)
V2
(W6)
| | Screening (V0) | Inclusion (V1) | Follow-up (V2) | Final (V3) |
| --- | --- | --- | --- | --- |
| Clinical interview | X | X | X | X |
| Blood pressure and anthropometry | X | X | X | X |
| Magnetic ressonance | | X | | X |
| Blood sample | X | X | | X |
| Stool collection | | X | | X |
| Dietary assessment | | X | | X |
| Physical activity assessment | X | X | X | X |
Supporting Figure S1

## Slide 2
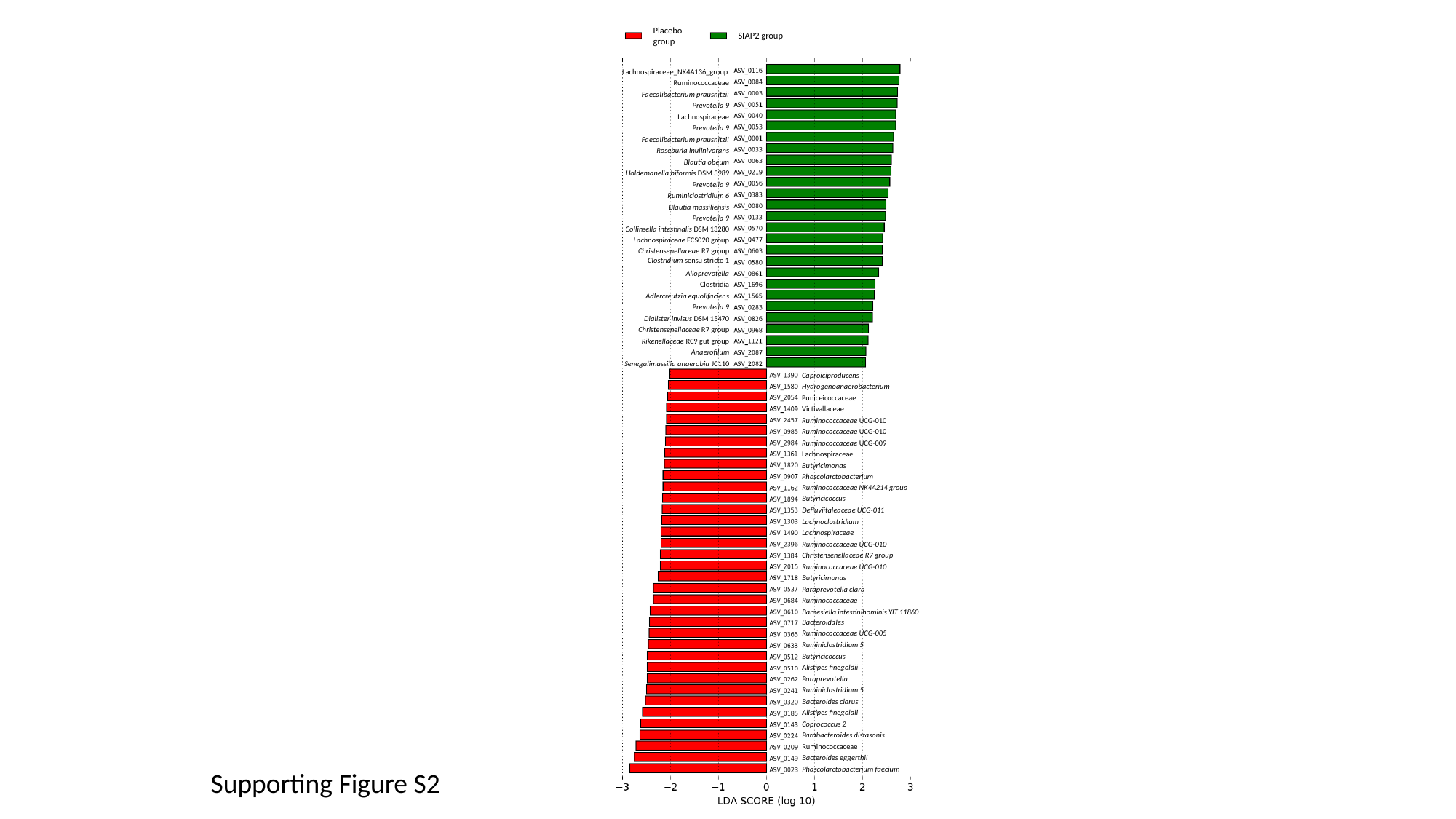

Placebo group
SIAP2 group
Lachnospiraceae_NK4A136_group
Ruminococcaceae
Faecalibacterium prausnitzii
Prevotella 9
Lachnospiraceae
Prevotella 9
Faecalibacterium prausnitzii
Roseburia inulinivorans
Blautia obeum
Holdemanella biformis DSM 3989
Prevotella 9
Ruminiclostridium 6
Blautia massiliensis
Prevotella 9
Collinsella intestinalis DSM 13280
Lachnospiraceae FCS020 group
Christensenellaceae R7 group
Clostridium sensu stricto 1
Alloprevotella
Clostridia
Adlercreutzia equolifaciens
Prevotella 9
Dialister invisus DSM 15470
Christensenellaceae R7 group
Rikenellaceae RC9 gut group
Anaerofilum
Senegalimassilia anaerobia JC110
Caproiciproducens
Hydrogenoanaerobacterium
Puniceicoccaceae
Victivallaceae
Ruminococcaceae UCG-010
Ruminococcaceae UCG-010
Ruminococcaceae UCG-009
Lachnospiraceae
Butyricimonas
Phascolarctobacterium
Ruminococcaceae NK4A214 group
Butyricicoccus
Defluviitaleaceae UCG-011
Lachnoclostridium
Lachnospiraceae
Ruminococcaceae UCG-010
Christensenellaceae R7 group
Ruminococcaceae UCG-010
Butyricimonas
Paraprevotella clara
Ruminococcaceae
Barnesiella intestinihominis YIT 11860
Bacteroidales
Ruminococcaceae UCG-005
Ruminiclostridium 5
Butyricicoccus
Alistipes finegoldii
Paraprevotella
Ruminiclostridium 5
Bacteroides clarus
Alistipes finegoldii
Coprococcus 2
Parabacteroides distasonis
Ruminococcaceae
Bacteroides eggerthii
Phascolarctobacterium faecium
Supporting Figure S2

## Slide 3
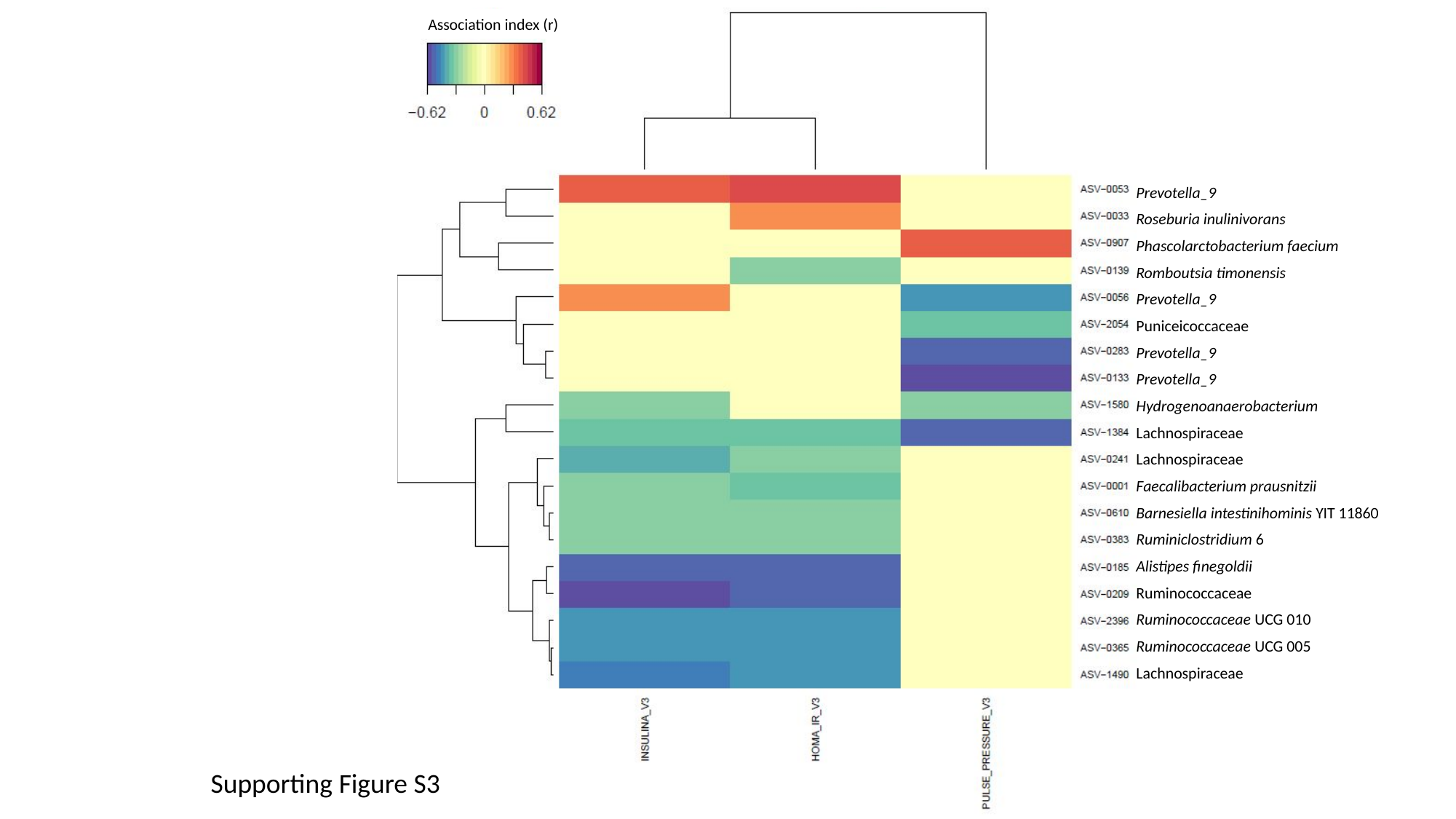

Association index (r)
Prevotella_9
Roseburia inulinivorans
Phascolarctobacterium faecium
Romboutsia timonensis
Prevotella_9
Puniceicoccaceae
Prevotella_9
Prevotella_9
Hydrogenoanaerobacterium
Lachnospiraceae
Lachnospiraceae
Faecalibacterium prausnitzii
Barnesiella intestinihominis YIT 11860
Ruminiclostridium 6
Alistipes finegoldii
Ruminococcaceae
Ruminococcaceae UCG 010
Ruminococcaceae UCG 005
Lachnospiraceae
Supporting Figure S3

## Slide 4
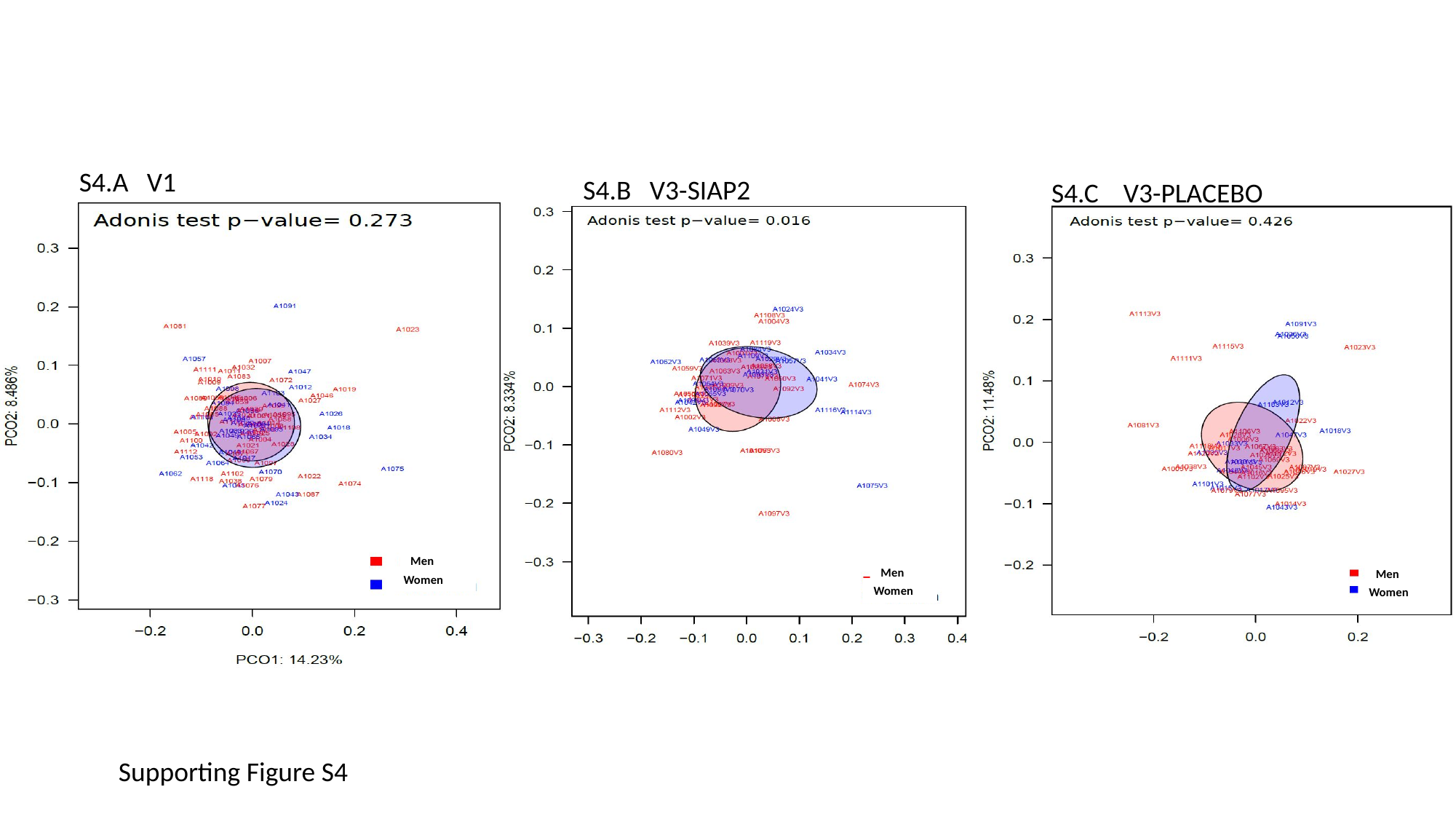

S4.A V1
S4.B V3-SIAP2
S4.C V3-PLACEBO
S4.A V1
Men
Men
Men
Women
Women
Women
Supporting Figure S4
